# Supplementary material for: Capping protein-controlled actin polymerization shapes lipid membranes
Source: Nat Commun. 2018 Apr 24;9:1630. doi: 10.1038/s41467-018-03918-1 (PMC5915599; doi:10.1038/s41467-018-03918-1)
Supplement: Supplementary file 1 — Supplementary Information [file 41467_2018_3918_MOESM1_ESM.pdf]

Supplementary Information

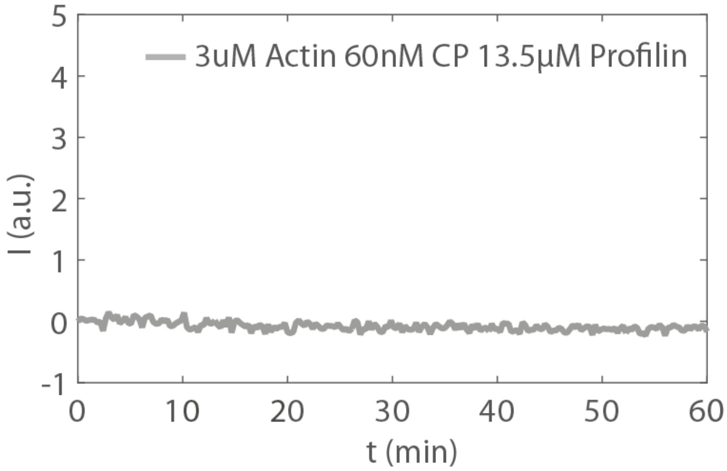

Supplementary Figure 1: The pyrene assays shows, that actin polymerization was inhibited for at least 60 min in the presence of 60 nM CP and 13.5 µM profilin.

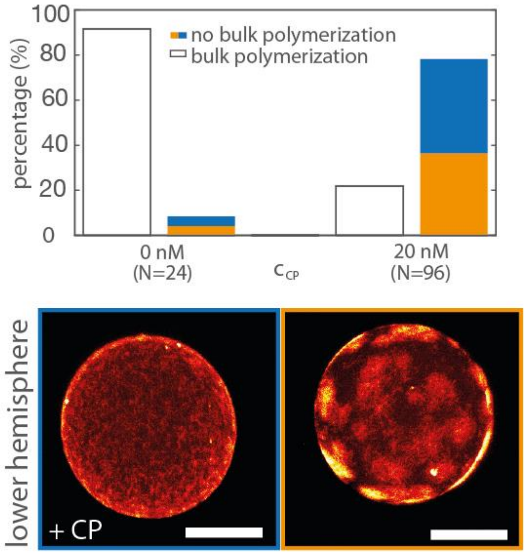

Supplementary Figure 2: Actin polymerization was fully shifted towards the membrane in the presence of 20 nM CP. Bulk polymerization was suppressed in 78% of the vesicles and only membrane binding was observed (orange-blue bar). Two binding modes occurred, which can be seen in the confocal z-projections of the lower vesicle's hemispheres. In 42% of the vesicles a homogeneous cortex (blue) and in 36% distinct domain formation (orange) could be observed at the membrane. We evaluated 24 vesicles at 0 nM CP and 96 vesicles at 20 nM CP. Scale bars are 20 µm.

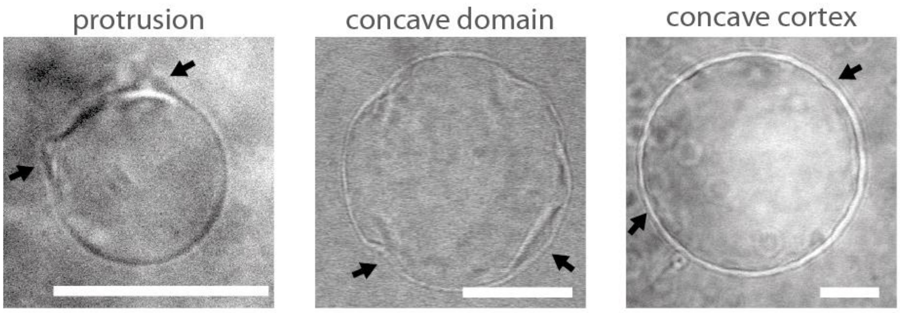

Supplementary Figure 3: Brightfield images of the vesicle's membrane are shown. The observed shape deformations of the membrane were induced by membrane localized actin polymerization. Actin protrusions pushed the membrane forward and created a membrane protrusion. Concave domains or concave cortices created negative curvatures within the membrane and bent it inwards. Scale bars are 20 µm.

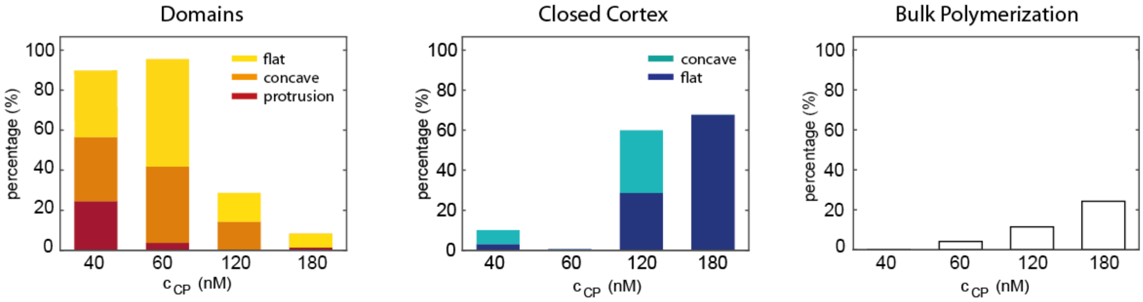

Supplementary Figure 4: A quantitative analysis of the appearing shape deformations dependent on the CP concentration is shown: The analysis was split in three subgroups: domains, closed cortices and bulk polymerization. We found three types of domain formation: flat domains (yellow), which did not deform the membrane, concave domains (orange), which bend the membrane inwards and protrusions (red), which pushed the membrane forward and induced a positive curvature of the membrane. The appearance of closed cortices at the vesicle's membrane could be split into two subgroups: concave cortices (cyan), which induced negative curvature within the membrane and flat cortices (blue), which did not deform the membrane. An increased polymerization of actin inside the vesicles (bulk polymerization) was observed at higher CP concentrations. Histogram counts are N= 159, 218, 70, 74 at c(CP) = 40, 60, 120, 180 nM respectively.

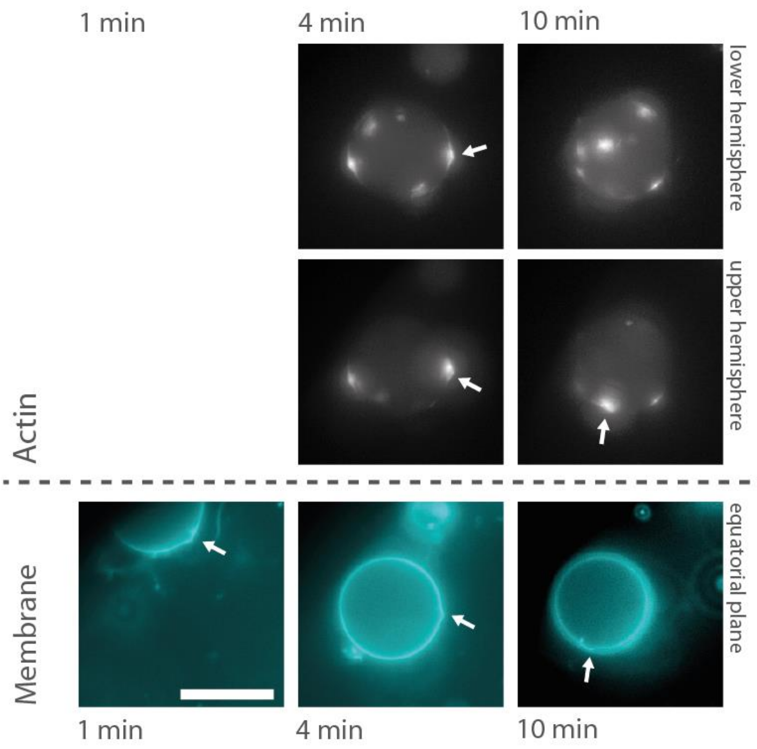

Supplementary Figure 5: Epifluorescence z-projections of snapshots at different time points are shown. The formation of actin protrusions and its resulting membrane protrusion was fast. The membrane protrusion was visible one minute after the production of vesicles was finished. Scale bar is 20 µm.

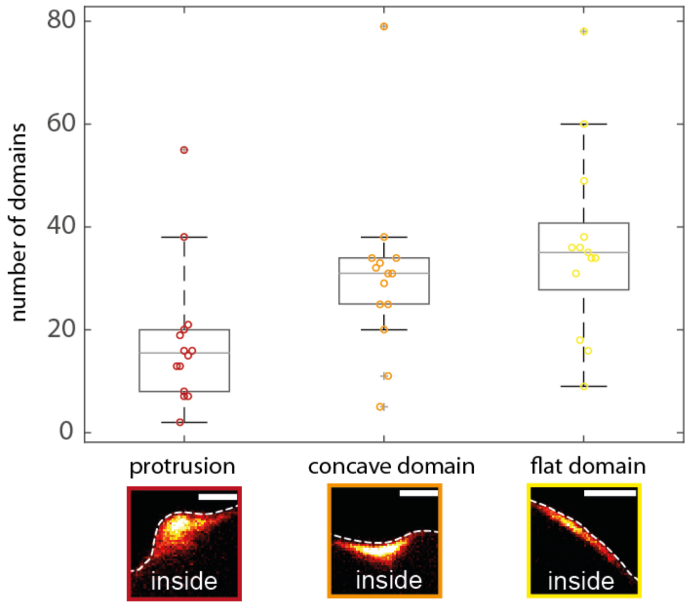

Supplementary Figure 6: The number of domains per vesicle was quantified for every vesicle type (either presenting protrusions, concave domains or flat domains). The resulting boxplots are shown: The median of the patch number per vesicle correlated with the observed domain shape. Medians were 15.5 for protrusions, 31 for concave domains and 35 for flat domains. Bottom panels illustrate the evaluated deformation types. The central grey line in the boxplots indicates the median. The bottom and top edges of the box mark the 25th and 75th percentiles, respectively. The whiskers got to the most extreme considered data point. Not considered data points are marked by a cross in the centre of the data point. Scale bars are 3 µm.

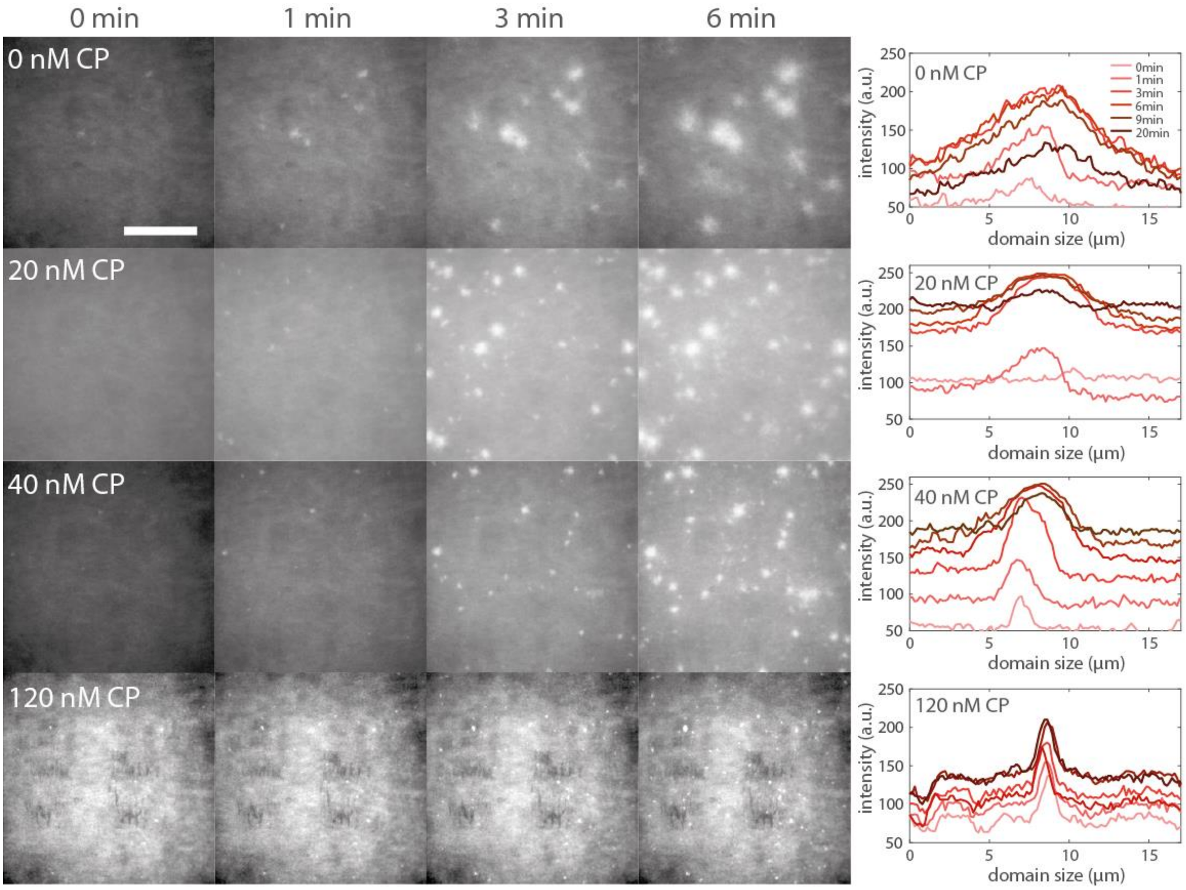

Supplementary Figure 7: The temporal evolution of actin domain growth on a lipid monolayer was investigated dependent on CP. On the left side TIRF-images at different time points show that speed and area of domain growth was controlled by CP. This observation was confirmed by measuring the intensity profile of one domain at six different time points at each CP concentration on the right side. Scale bar is 20µm.

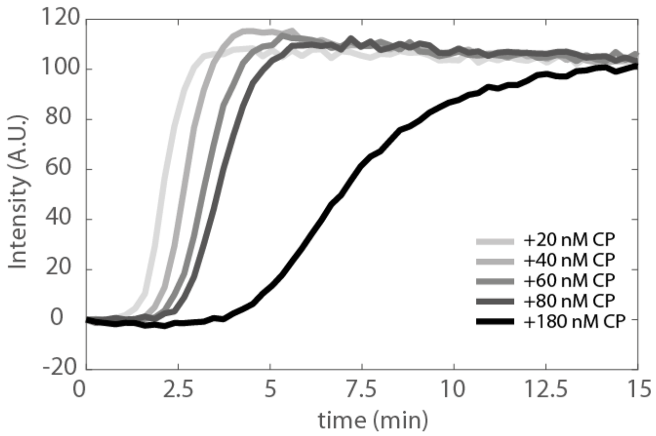

Supplementary Figure 8: The polymerization kinetics of branched actin networks (3 µM actin, 0.3 µM VCA, 0.3 µM Arp2/3 complex and 13.5 µM profilin) were measured in pyrene assays depended on the concentration of CP. The lag time before polymerization initiation increased and the polymerization speed after initiation of actin assembly decreased with an increasing CP concentration.

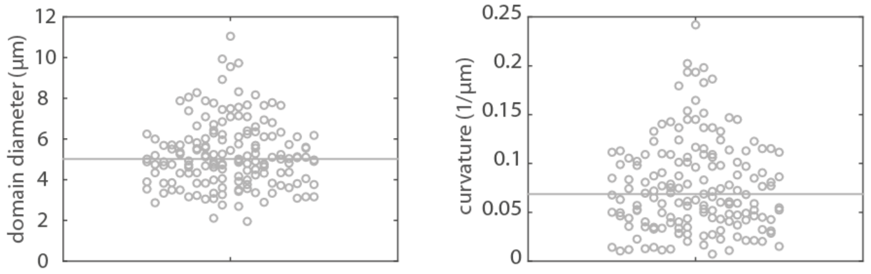

Supplementary Figure 9: The size distribution of actin domains and its curvature at 40nM CP is shown. The median length was about 5 µm and the median curvature 0.07 µm^{-1}.

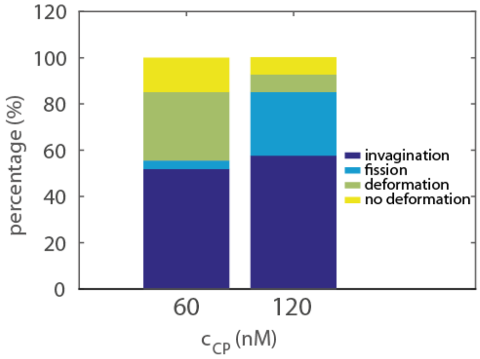

Supplementary Figure 10: The quantification of the contractile response of non-muscle myosin II is shown. It was dependent on the concentration of CP. 3 µM actin, 0.3 µM VCA, 0.3 µM Arp2/3 complex, 13.5 µM profilin and 5 µM non-muscle myosin II were encapsulated inside of vesicles in the presence either of 60 nM CP or 120 nM CP. This resulted not only in the formation of local membrane deformations but also in the formation of membrane invaginations, which (navy) were visible in more than 50% of the vesicles at both CP concentrations. However, fission events (detached membrane invaginations, blue) occurred preferably at 120 nM CP. The histogram counts at 60 nM CP are 27 and at 120 nM CP are 40 vesicles. At least two independent experiments per CP concentration were performed.

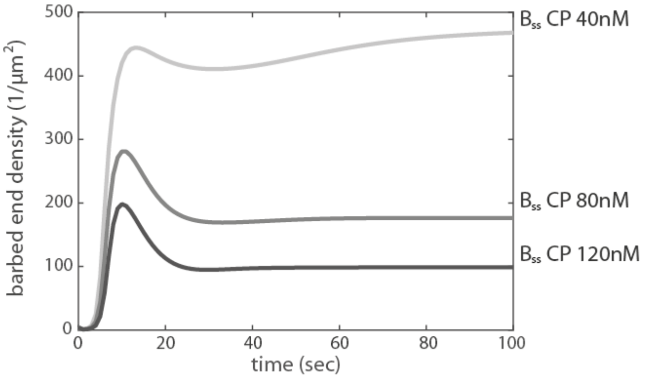

Supplementary Figure 11: The barbed end density is modelled over time at different CP concentrations. The steady state was reached for all CP concentrations. The final steady-state level depended on the concentration of CP after 100 sec.

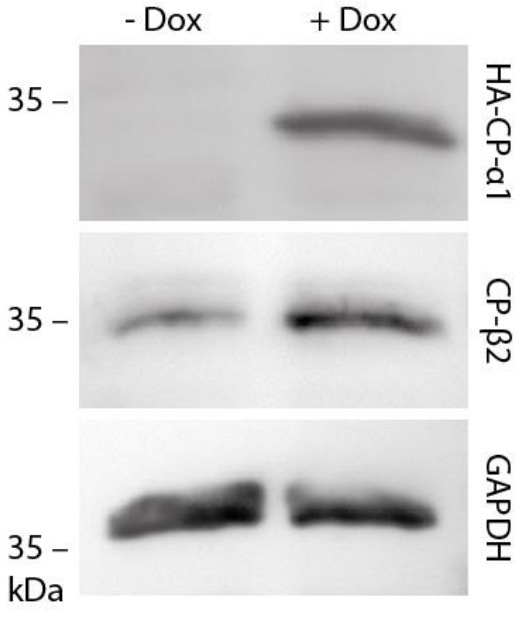

Supplementary Figure 12: Relative levels of CP in B16-F1 cells before and after induction with 1 µM Doxycyclin. 24 h post transfection, the cells were harvested, boiled in SDS sample buffer and the proteins resolved by SDS-PAGE. After transfer to nitrocellulose the membrane was stained with the antibodies indicated. Note lack of HA-CP-α1 expression in the absence of Doxycyclin and increase of CP-ß2 in the presence of Doxycyclin. The relative average amount of CP was quantified by densitometry of immunoblots labelled with CP beta antibodies. Immunostaining with GAPDH served as a loading control.

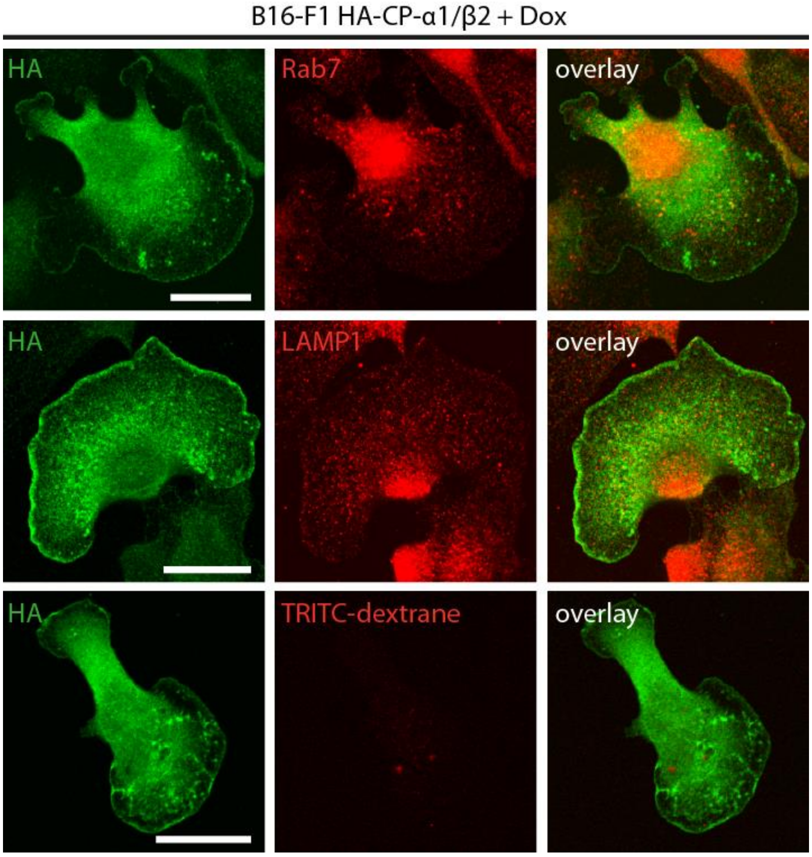

Supplementary Figure 13: The majority of CP-containing vesicles (green) in the overexpressing cells did not colocalize with the endosomal marker Rab7 and the lysosomal marker LAMP1 (red). These vesicles also did not display any enrichment of the fluid-phase marker TRITC-dextrane (red) excluding the likelihood of being enclosed and internalized by membrane ruffling through macropinocytosis. The scale bars are 20 µm.

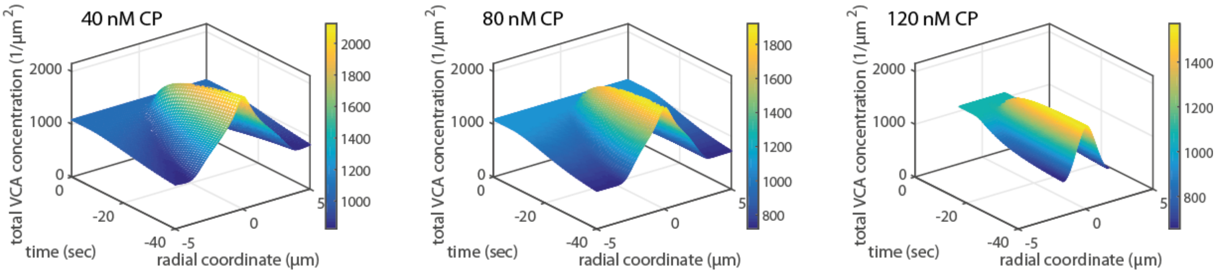

Supplementary Figure 14: The spatio-temporal evolution of the total number of membrane bound VCA molecules (V+V*+A*+V^+) at finite protein pools is modelled at three different CP concentrations (40 nM, 80 nM and 120 nM). We defined a "paused-state" (see Supplementary Notes) for VCA to implement effectively the experimentally observed VCA up-concentration in the simulations. It can be seen, that VCA concentrates within the growing actin domain for all CP concentrations, as the VCA surface density is high in the domain center and decreases towards the domain edges.

Supplementary Note 1

Kinetic model simulations of network growth

We model the growth of the actin network by implementing the kinetic equations into a system of differential equations which is solved numerically in three dimensions using finite elements in COMSOL Multiphysics 5.3 (COMSOL Inc.). Thereby we follow previous models for cellular lamellipodial growth[1,2], that can be simplified for our limited set of proteins: Actin, Arp2/3 complex, CP and VCA. Due to the lack of the actin depolymerization machinery we assume the network to be passive once it is formed and model activity only at the network front at the membrane. Additionally, we extend the model to include the VCA activator system[3,4]. Furthermore, we include diffusion and take into account the finite volume of the vesicle. From the protein binding kinetics, we derive the chemical composition of the network neglecting mechanical interactions with the membrane. The identified effect on the barbed end density discussed in the main text is also observable in a model neglecting finite vesicle volume and diffusion effects, which is not presented in this work.

Kinetic equations

$$\text{Nucleation:} \quad \mathrm{G} + \mathrm{G} \rightleftharpoons \mathrm{G_2}; \quad \mathrm{G_2} + \mathrm{G} \rightleftharpoons \mathrm{G_3} := \mathrm{F_3}; \tag{1}$$

$$\text{Polymerization:} \quad \mathrm{F_n} + \mathrm{G} \underset{k_{off}}{\overset{k_{on}}{\rightleftharpoons}} \mathrm{F_{n+1}}; \tag{2}$$

$$\text{Arp2/3-Activation:} \quad \mathrm{VCA} + \mathrm{G} \underset{k_2}{\overset{k_1}{\rightleftharpoons}} \mathrm{VCAG}; \quad \mathrm{VCAG} + \mathrm{Arp2/3} \underset{k_4}{\overset{k_3}{\rightleftharpoons}} \mathrm{Arp^*}; \tag{3}$$

$$\text{Branching:} \quad \mathrm{Arp^*} + \mathrm{F_n} \xrightarrow{k_{Arp}} \mathrm{F_n} + \mathrm{F_1} + \mathrm{VCA}; \tag{4}$$

$$\text{Capping:} \quad \mathrm{CP} + \mathrm{F_n} \xrightarrow{k_{Cap}} \mathrm{CPF_n}; \tag{5}$$

With the notation:

$\mathrm{G/G_2/G_3}$	actin monomer/dimer/trimer
$\mathrm{F_n}$	actin filament of n subunits
VCA	Arp2/3-activating region of WASP family proteins
VCAG	complex of VCA with an actin monomer (activated VCA)
$\mathrm{Arp^*}$	complex of Arp2/3 with VCAG (activated Arp)
CP, $\mathrm{CPF_n}$	Capping Protein, capped actin filament

Where the k_i denoting the respective reaction rates.

Supplementary Note 2

Implementation of the model

As only a few domains are present in a vesicle and most of them are similar in shape we can assume that our observed actin domains originate from single seeds. Thus, we do not include the spontaneous actin nucleation process (equation (1)) in our kinetic equations.

We neglect depolymerization on the filament pointed ends in equation (2) as we consider them to be stabilized by Arp2/3. The Arp2/3 can only bind to a filament (equation (4)), after it has been activated by VCA on the membrane (equation (3)). We neglect unbinding of CP and debranching, as we assume these processes to take place on a slower time scale.

The model implements a system of differential equations for the surface densities of VCA (V), VCA-actin complex VCAG (V^*), an optional paused state for VCA (V^+) (see below) activated Arp2/3 complex (A^*) and the barbed end density (B). The binding interaction of Arp2/3 and actin is proportional to the number of filaments. The number of filaments at the membrane is equal to the number of barbed ends.

Geometry

We describe the system as cylindrically symmetric. The simulated geometry is a cone shape that represents the volume element of the vesicle assigned to a single domain (simulated by a cylindrically symmetric triangle). Its circular disc describes the membrane and the tip of the cone is the centre of the vesicle. The size of the cone is chosen according to the experimental data, where the height is the average vesicle radius (18 µm). As the cone describes the volume element for one domain, its circular disk has to be half the centre-to-centre distance between two domains. This value depends on the CP concentration, as we observe significantly different numbers of domains. We use 10 µm at 40 nM CP (16 domains), 6 µm at 80 nM CP (35 domains) and 1.7 µm at 120 nM CP (measured directly in image data). We set von-Neumann zero flux conditions at the surface boundary.

In the case to model infinite pools, we use a cylinder geometry (r=7 µm, h= 9µm). At the side opposing the membrane, the solution concentrations (G, Arp, CP) obey fixed Dirichlet boundary condition keeping their initial value.

Equations

We separate the system into the membrane bound species (VCA, VCAG, A^*, B, V^+) that are simulated in 2d, and the solution concentrations (G, CP, Arp) that are simulated in 3d. We include 3d diffusion for G, CP & Arp and 2d diffusion for V, V^* & A^*. B also gets a diffusive term describing the spreading of the network, which is discussed below.

Membrane bound 2d species:

$$\frac{\partial V}{\partial t} = D_V \nabla_{\mathrm{m}}^2 V - k_1 G|_0 V + k_2 V^* + k_{\mathrm{re}} V^+ \tag{6}$$
$$\frac{\partial V^*}{\partial t} = D_{V^*} \nabla_{\mathrm{m}}^2 V^* + k_1 G|_0 V - k_2 V^* - k_3 Arp|_0 V^* + k_4 A^* \tag{7}$$
$$\frac{\partial A^*}{\partial t} = D_A \nabla_{\mathrm{m}}^2 A^* + k_3 Arp|_0 V^* - k_4 A^* - k_{\mathrm{Arp}} A^* B \tag{8}$$
$$\frac{\partial B}{\partial t} = D_B G|_0 / G_{\mathrm{init}} \nabla_{\mathrm{m}}^2 B + k_{\mathrm{Arp}} A^* B - k_{\mathrm{Cap}} CP|_0 B - k_{\mathrm{Deg}} B \tag{9}$$
$$\frac{\partial V^+}{\partial t} = k_{\mathrm{Arp}} A^* B - k_{\mathrm{re}} V^+ \tag{10}$$

where $|_0$ denotes the concentration of cytosolic species at the membrane and ∇_{m}^2 denotes the diffusion operator at the membrane.

Cytosolic 3d species:

Because all reactions happen at the membrane, they lead to fluxes in the cytosol, in the direction normal to the membrane. In equations for the cytosolic species these fluxes do not show up in the bulk dynamics directly

$$\frac{\partial G}{\partial t} = D_{\mathrm{G}} \nabla^2 G \tag{11}$$
$$\frac{\partial Arp}{\partial t} = D_{\mathrm{Arp}} \nabla^2 Arp \tag{12}$$
$$\frac{\partial CP}{\partial t} = D_{\mathrm{CP}} \nabla^2 CP \tag{13}$$

But appear as boundary conditions

$$-D_{\mathrm{G}} \partial_z G|_{z=0} = -k_1 G|_0 V - k_{\mathrm{on}} G|_0 B + k_{\mathrm{off}} B \tag{14}$$
$$-D_{\mathrm{Arp}} \partial_z Arp|_{z=0} = -k_3 Arp|_0 V^* \tag{15}$$
$$-D_{\mathrm{CP}} \partial_z CP|_{z=0} = -k_{\mathrm{Cap}} CP|_0 B \tag{16}$$

where $\partial_z \ldots |_{z=0}$ denotes the derivative in normal direction to the membrane, evaluated at the membrane (z=0).

Kinetic rates

For the interaction constants we take literature values: k_{on}=11.6 /µM/s and k_{off}=1.4 /s from[5]; k_1=43 /µM/s, k_2=30 /s, k_3=0.8 /µM/s and k_4=0.6 /s from[3]; k_{Cap}=4 /µM/s[6]. We reduced k_3 by a factor of 3.3 to better fit our experimental results. The interaction parameter for branching k_{Arp}=0.02 µm²/s is set such that steady network growth is possible.

Diffusion constants

We use the literature value D_{G}=30 µm²/s for actin[7] and D_{V}=1.59 µm²/s, determined by FRAP. The other diffusion constants are scaled from these values according to the Einstein-Stokes equation, approximating them as globular proteins (D_{CP}=26 µm²/s, D_{Arp}=17 µm²/s, D_{V}=0.96 µm²/s, D_{A}=0.84 µm²/s).

Initial values

We set the initial solution concentrations to G=3 µM, Arp2/3=0.3 µM and CP according to the experiment. The initial value for the barbed ends B_0(r,0) is set to be a Gaussian distribution around r=0 with σ=10 nm normalized to 1/µm^2 to describe the seeding event. The density of VCA is set to V_0=1084 /µm^2, assuming all VCA bound homogeneously to the membrane and a vesicle radius of 18 µm and $V^*=A^*$=0 /µm^2.

Spreading of the actin network

Little is known about the spreading of the branched actin network along the membrane and thus we choose a simplistic description. We assume that the branching process can be regarded as a random walk. As the spatial growth for the network can be identified by the filament ends, this random walk is described by a diffusive term for density B with constant D_B. As lateral growth should be proportional to filament elongation, the term is further scaled by the local actin monomer concentration $G|_0$ normalized by its initial value G_{init}. In the simulation we find an approximately linear growth regime in r-direction, despite the assumption of diffusive branching and spreading process (Fig. 5c). This is due to the self-amplifying network growth process. As the spreading behaviour of real networks is unknown, the constant D_{B}=0.0077 µm^2/s is chosen to return spreading speeds in the range of the filament growth velocity, that also fit the observed final domain sizes.

Paused state of VCA

We add a paused state for VCA upon branch creation, to take account for the concentration of VCA in the domains, which we observed in the experimental data. In this paused state we consider VCA to be immobile (V^+) and not available for a new activation by G-actin monomer binding. With a rate k_{re}, VCA returns into its ground state V. We set k_{re} to 0.001 /s to fit the experimental finding of an approximately 4-fold higher VCA concentration in the final domain compared to its environment (see Supplementary Figure 14). For the studies at infinite protein pools we neglected the introduction of a paused state and enhanced k_{re} to 1000 /s to ensure that CP controlled reaction kinetics do not rely on the VCA up-concentration. Also, at finite protein pools CP dependent pattern formation could be observed even without the implementation of the paused state. The comparison between simulations at finite protein pools in the presence and absence of the paused state show that it only enhances the concave character of the domains, as it depletes the activator. We would like to point out that pattern formation does not rely on the up-concentration of VCA. The here used paused state of VCA is one suggestion to implement VCA up-concentration, that may have its origin in immobilization due to filament binding or reduced diffusivity due to molecular crowding.

Barbed end degradation term

To enhance numerical stability, we also include a degradation term for the barbed end density. Such a term also accounts for filaments that stay behind and do no longer reach the membrane for further branching. We choose a small rate k_{Deg}=0.001 /s, that is comparable to 1 nM CP and thus comes only into play if the local CP concentration is depleted.

Supplementary Methods

Preparation of lipid monolayers

The monolayers were prepared according to the protocol provided by ibidi. At first the desired lipid stored in chloroform (DOPC (Avanti Lipids, 850375), 10% Ni-NTA (Avanti Lipids, 790404C) and 2.5% PEG 2000 PE (Avanti Lipids, 880160C)) were mixed in a glass vial. The total amount of lipids was 1 mg. The chloroform was evaporated under the hood and the glass vial was stored under vacuum for several hours to remove any remaining traces of chloroform. Afterwards the lipids were dissolved in a mixture of 70%/30% ddH$_2$O and isopropanol and vortexed for 1 min. More isopropanol should me added if the mixture is not clear. 50 µl of the lipid mix were filled into a channel of an µ-Slide VI$^{0.4}$ uncoated (ibidi, 80606). The channel was washed slowly with 1 ml ddH$_2$O and rinsed quickly a few times with water to remove any traces of isopropanol.

Polymerization of branched networks on lipid monolayers

The monolayers were washed with VCA buffer (20 mM Tris/HCl, 100 mM KCl, 1 mM MgCl$_2$, 5 mM EGTA, 2 mM DTT, pH 7.0) and incubated with 300 nM His-VCA. After 10 min the channels were extensively washed with VCA buffer to remove any unbound VCA. To prepare the branched network polymerization mix 3 µM actin, 0.3 µM Arp2/3 complex, 13.5 µM profilin and the desired amount of CP were added to polymerization buffer (10 mM imidazole, 3 mM MgCl$_2$, 30 mM KCl, 1 mM EGTA, pH 7.4). The mix was filled into the channels and imaging could be started immediately. To image the network growth 10% of unlabelled actin were replaced by Atto 488 actin. The polymerization mix was supplemented with 0.4% glucose, 0.18 mg/ml glucose-oxidase and 0.06 mg/ml catalase to minimize photobleaching of Atto 488. The sample was imaged at room temperature on a commercially built TIRF microscope (Leica AF6000 Modular Systems, Leica). Images were taken with an iXon Ultra EMCCD camera (Andor).

Supplementary References

1. Mogilner, A. & Edelstein-Keshet, L. Regulation of Actin Dynamics in Rapidly Moving Cells. A Quantitative Analysis. *Biophysical Journal* **83,** 1237–1258 (2002).
2. Tania, N., Condeelis, J. & Edelstein-Keshet, L. Modeling the synergy of cofilin and Arp2/3 in lamellipodial protrusive activity. *Biophysical Journal* **105,** 1946–1955 (2013).
3. Marchand, J. B., Kaiser, D. A., Pollard, T. D. & Higgs, H. N. Interaction of WASP/Scar proteins with actin and vertebrate Arp2/3 complex. *Nat Cell Biol* **3,** 76–82 (2001).
4. Achard, V. *et al.* A "primer"-based mechanism underlies branched actin filament network formation and motility. *Curr Biol* **20,** 423–428 (2010).
5. Pollard, T. D., Blanchoin, L. & Mullins, R. D. Molecular mechanisms controlling actin filament dynamics in nonmuscle cells. *Annu Rev Biophys Biomol Struct* **29,** 545–576 (2000).
6. Schafer, D. A., Jennings P. B., Cooper J. A. Dynamics of capping protein and actin assembly in vitro. Uncapping barbed ends by polyphosphoinositides. *J Cell Biol* **135,** 169–179 (1996).
7. Lanni, F. & Ware, B. R. Detection and characterization of actin monomers, oligomers, and filaments in solution by measurement of fluorescence photobleaching recovery. *Biophysical Journal* **46,** 97–110 (1984).
